# The triglyceride-glucose index as a predictor of cardiovascular disease: an umbrella review of meta-analyses

**DOI:** 10.1097/MS9.0000000000005559

**Published:** 2026-08-27

**Authors:** Shahriar Ghodous, Herby Jeanty, Saisree Reddy Adla Jala, Kavya Priya Somu, Nikitha Chellapuram, Shika M Jain, Pegah Rashidian, Abinash Mahapatro, Maryam Jafari, Seyedsina Moghimnejadhosseini, Hoda Safa, Mohammad Hashemi, Arezoo Ghazalgoo

**Affiliations:** a School of Medicine, Guilan University of Medical Sciences, Rasht, Iran; b The Brooklyn Hospital Center, USA; c Mission Hospital, Asheville, NC, USA; d Centinela Hospital Medical Center 555 E Hardy St, Inglewood, CA, USA; e Cooper University Hospital, Camden, NJ, USA; f MVJ Medical College and Research Hospital, Bengaluru, India; g Vali-e-Asr Reproductive Health Research Center, Family Health Research Institute, Tehran University of Medical Sciences, Tehran, Iran; h SIU School of Medicine, Springfield, IL, USA; i Faculty of Medicine, Semmelweis University, Budapest, Hungary; j Department of Endocrinology, Endocrinology and Metabolism Research Center, Hormozgan University of Medical Sciences, Bandar Abbas, Iran; k Cardiovascular Research Center, Hormozgan University of Medical Sciences, Bandar Abbas, Iran

**Keywords:** cardiovascular disease, insulin resistance, meta-analysis, risk assessment, triglyceride-glucose index

## Abstract

**Background:**

The triglyceride-glucose (TyG) index has gained recognition as a tool for assessing cardiovascular disease risk. This umbrella review synthesized findings from multiple meta-analyses examining the association between the TyG index and cardiovascular outcomes.

**Methods:**

In this umbrella review of meta-analyses, we followed the Preferred Reporting Items for Systematic Reviews and Meta-Analyses (PRISMA) guidelines.

**Results:**

A higher TyG index was associated with increased odds of arterial stiffness [categorical: odds ratio (OR) = 1.83, 95% confidence interval (CI): 1.55–2.17, *P* < 0.01; *I*^2^ = 68%; per 1-unit increase: OR = 1.51, 95% CI: 1.35–1.69, *P* < 0.01; *I*^2^ = 82%]; coronary artery stenosis (OR = 3.49, 95% CI: 1.71–7.12, *P* < 0.01; *I*^2^ = 0%); coronary artery calcification (CAC) (highest vs. lowest: OR = 1.66, 95% CI: 1.51–1.82, *P* < 0.01; *I*^2^ = 0%; continuous: OR = 1.73, 95% CI: 1.36–2.20, *P* < 0.01; *I*^2^ = 51%); CAC progression (categorical: OR = 1.66, 95% CI: 1.21–2.27, *P* < 0.01; *I*^2^ = 0%; continuous: OR = 1.47, 95% CI: 1.29–1.68, *P* < 0.01; *I*^2^ = 41%); and atrial fibrillation (AF) (categorical: OR = 1.98, 95% CI: 1.35–2.91, *P* < 0.01; *I*^2^ = 92%; continuous: OR = 1.80, 95% CI: 1.50–2.17, *P* < 0.01; *I*^2^ = 88%).

**Conclusion:**

Current meta-analytic evidence suggests that higher TyG index values are associated with several adverse cardiovascular outcomes. However, substantial heterogeneity and the generally low methodological quality of the included meta-analyses limit confidence in the magnitude and clinical applicability of these associations. The TyG index may serve as an adjunctive cardiovascular risk marker, but further high-quality prospective validation is required before routine clinical implementation.

## Introduction

The triglyceride-glucose (TyG) index, derived from routine measurements of fasting triglycerides and fasting glucose, has emerged as a practical and reliable surrogate marker of insulin resistance (IR)^[^1–4^]^. IR plays a fundamental pathophysiological role in the development and progression of cardiovascular disease (CVD), primarily through its effects on endothelial dysfunction, atherogenic dyslipidemia, and chronic low-grade inflammation^[^5–7^]^. Unlike conventional individual metabolic parameters, such as isolated glucose or lipid levels, the TyG index offers a more integrated reflection of the underlying metabolic disturbances that collectively drive cardiovascular risk[8].

HIGHLIGHTS

- This umbrella review consolidates 27 meta-analyses to demonstrate a strong association between an elevated triglyceride-glucose (TyG) index and a wide range of cardiovascular diseases (CVDs), including hypertension, coronary artery disease, and heart failure.
- The TyG index, a simple and cost-effective biomarker, shows promise for cardiovascular risk stratification, particularly in resource-limited settings where advanced testing may be unavailable.
- Elevated TyG levels are consistently linked to a higher risk of myocardial infarction, arterial stiffness, and cardiovascular mortality, emphasizing their role in the early detection and prevention of CVD.

CVD remains the leading cause of morbidity and mortality worldwide, underscoring the urgent need for accessible, cost-effective, and accurate risk stratification tools^[^9–11^]^. Over the past decade, a growing body of research has examined the relationship between the TyG index and various cardiovascular outcomes (CVOs), including coronary artery disease (CAD), stroke, heart failure (HF), and adverse events following revascularization procedures^[^12–14^]^. Most of these studies suggest that an elevated TyG index is associated with heightened cardiovascular risk, likely because it captures the cumulative metabolic burden imposed by IR on the vascular wall and during atherosclerotic progression[15].

Despite the consistency of this overall direction of association, individual studies have reported considerable variability in the magnitude of associations, partly due to differences in study populations (e.g., general populations versus high-risk clinical cohorts), varying TyG cutoff values, divergent outcome definitions, and heterogeneous follow-up durations. Such variability complicates the translation of these findings into unified clinical recommendations. To address these inconsistencies and synthesize the expanding body of evidence, numerous meta-analyses have been conducted in recent years^[^14,16,30,25^]^. These quantitative syntheses have provided more precise estimates of the association between the TyG index and specific cardiovascular endpoints and have explored sources of heterogeneity across primary studies. Meta-analyses represent a higher level of evidence than individual observational studies, as they increase statistical power and enable systematic exploration of between-study differences.

Although numerous individual meta-analyses have examined the association between the TyG index and various CVOs, their findings are dispersed across different publications, populations, and outcome definitions. This fragmentation makes it difficult for clinicians and researchers to obtain a comprehensive, integrated picture of the current evidence. Therefore, an umbrella review is needed to systematically aggregate, synthesize, and critically appraise all available meta-analyses on this topic. Accordingly, the present umbrella review aims to comprehensively summarize the existing meta-analyses and provide a unified, high-level overview of the current evidence regarding the TyG index and CVOs.

## Methods

### Study design

This umbrella review consolidates evidence from meta-analyses investigating the association between the TyG index and various CVOs. To ensure a systematic and transparent approach, we adhered to the Preferred Reporting Items for Systematic Reviews and Meta-Analyses (PRISMA) guidelines throughout the review process[18]. The study protocol was registered in PROSPERO (CRD42024564660).

### Search strategy

A comprehensive literature search was conducted across PubMed, Scopus, and Web of Science to identify relevant meta-analyses published up to October 2025. The search strategy combined keywords and Medical Subject Headings terms related to the TyG index, CVOs, and meta-analysis. The complete search strategy is provided in the Supplemental Digital Content Table S1, available at: https://links.lww.com/MS9/B352. Two independent reviewers screened titles and abstracts for eligibility, after which full-text articles were retrieved for further assessment.

### Inclusion and exclusion criteria

Meta-analyses examining the relationship between the TyG index and any CVO were considered eligible. Included studies were required to report effect estimates, including odds ratios (ORs), relative risks (RRs) or hazard ratios (HRs) with 95% confidence intervals (CIs). Meta-analyses that did not specifically evaluate the TyG index or lacked quantitative synthesis (e.g., systematic reviews without meta-analysis) were excluded. Non-English publications, original studies, commentaries, editorials and letters were also excluded.

### Data extraction

Two reviewers independently extracted data from the included meta-analyses. Extracted information included the first author’s name, publication year, overall sample size, the CVO evaluated, effect estimates (ORs, RRs, or HRs) with 95% CIs, heterogeneity measures (*I*^2^), and the risk-of-bias assessment tools used in each meta-analysis. Disagreements during data extraction were resolved by consensus or through consultation with a third reviewer.

### Quality assessment

The methodological quality of each included meta-analysis was assessed using the AMSTAR-2 (A Measurement Tool to Assess Systematic Reviews 2) checklist. Based on AMSTAR-2 criteria, studies were classified as having “high,” “moderate,” “low,” or “critically low” quality[19]. The results of this assessment are presented in Table 1.

**Table 1:** Characteristics of the included studies.

First author	Country	Outcomes	Journal	Number of included studies	Number of included patients	Analysis software	Date of search	Databases searched	Protocol registration	Quality assessment	AMSTAR 2 score
Ding *et al*[31]	China	ASCVD, CAD	Cardiovascular Diabetology	8	5 731 298	RevMan (v:5.1) and STATA (v:12)	Up to 5 January 2021	PubMed, Embase	No	Newcastle–Ottawa Scale for cohort studies	Critically low
Lou *et al*^[^18^]^	China	MACE in CAD, MI in CAD, revascularization in CAD, CV death in CAD	Frontiers in Cardiovascular Medicine	12	28 795	STATA (v:12)	Up to 5 October 2021	PubMed, Scopus, EMBASE, and Web of Science	PROSPERO: CRD42021228521	The Newcastle-Ottawa-Scale for cohort and case-control studies	Low
Wang *et al*[22]	China	HTN in adults	Frontiers in Cardiovascular Medicine	8	200 044	RevMan (v:5.1) and STATA	Up to 10 March 2021	PubMed, Embase, Web of Science, Wanfang data, and Chinese National Knowledge Infrastructure (CNKI)	No	Newcastle-Ottawa Scale	Critically low
Liu *et al*[23]	China	CAD in general population, MI in general population, CVD in general population, CV death in general population	Cardiovascular Diabetology	12	6 354 990	RevMan (v:5.4)	Up to 16 April 2022	PubMed, Embase, and the Cochrane Library	PROSPERO: CRD42022296235	Newcastle-Ottawa-Scale for cohort studies	Critically low
Sajdeya *et al*[24]	USA	Arterial stiffness	Current Problems in Cardiology	9	37 780	RevMan (v:5.3)	Up to 30 April 2022	PubMed, Embase, and Web of Science	No	Newcastle Ottawa Quality Assessment Scale	Critically low
Chang *et al*[16]	Taiwan	CIN after PCI	Frontiers in Endocrinology	5	3518	RevMan (v:5.4)	Up to 3 August 2023	MEDLINE, Cochrane Library, and EMBASE	PROSPERO: CRD42023452257	Quality Assessment for Diagnostic Accuracy Studies-2 (QUADAS-2)	Low
Khalaji *et al*[14]	Iran	HF	Cardiovascular Diabetology	30	772 809	R (v: 4.3.0)	Up to 16 June 2023	PubMed, Embase, Scopus, and Web of Science databases	PROSPERO: CRD42023437470	Newcastle-Ottawa Scale	Critically low
Liang *et al*^[^12^]^	China	CAD, CAD severity, MACE in ACS, MACE in CCS or CAD,	Cardiovascular Diabetology	41	–	RevMan (v:5.3.5)	Up to 1 May 2023	PubMed, EMBASE, The Cochrane Library, and Web of Science databases	PROSPERO: CRD42023422917	Newcastle–Ottawa Scale	Critically low
Liu *et al*[25]	China	Arterial stiffness, CAC	Cardiovascular Diabetology	26	87 307	Stata 16.0	Up to 9 September 2022	PubMed, EMBASE, The Cochrane Library	PROSPERO: CRD42022360981	Newcastle Ottawa Quality Assessment Scale	Low
Lukito *et al*[26]	Indonesia	HTN	Narra J	14	108.396	STATA 17.0	Up to 1 June 2024	PubMed, Embase, Scopus, and ScienceDirect	PROSPERO: CRD42021235595	Newcastle-Ottawa Scale	Critically low
Xu *et al*[27]	China	HTN	Frontiers in Cardiovascular Medicine	22	668 486	Stata version 17.0, Rstudio version 4.2.1	Up to 10 May 2023	CNKI, VIP, Wanfang Data, CBM, PubMed, EMbase, Web of Science, and The Cochrane Library	PROSPERO: CRD42023425836	Agency for Healthcare Research and Quality (AHRQ) scale and Newcastle-Ottawa Quality Assessment Scale (NOS)	Low
Zhong *et al*[28]	China	Arterial stiffness	Medicine	13	54 327	RevMan (v:5.1) and STATA 14.0	Up to 31 July 2022	PubMed, Embase, and Scopus	PROSPERO: CRD42022330963	Newcastle–Ottawa Scale	Critically low
Azarboo *et al*[29]	Iran	Risk of AF, risk of post-procedural AF, risk of post-ablation AF	European Journal of Medical Research	9	886	R version 4.3.0	Up to September 2023	PubMed, Embase, Scopus, and Web of Science	PROSPERO: CRD42023456875	Newcastle–Ottawa Scale	Critically low
Jiang *et al*[17]	China	ISR in CHD, ISR in ACS, ISR in CCS	BMC Cardiovascular Disorders	5	3912	RevMan (v:5.4.1)	1990–2024	PubMed, EMBASE, Web of Science, and Cochrane Library databases	PROSPERO: CRD42023482588	Newcastle-Ottawa Scale	Critically low
Liu *et al*[30]	China	MACCE after AMI,	Nutrition & Diabetes	21	20 403	RevMan (v:5.4) and STATA 18.0	Up to 18 October 2023	PubMed, MEDLINE, Web of Science, Embase and Cochrane Library databases	No	Newcastle–Ottawa Scale	Critically low
Samavarchitehrani *et al*[32]	Iran	PAD, severity of PAD, diagnostic ability of TyG in PAD	Nutrition & Diabetes	22	73 168	R version 4.3.0	Up to April 2024	PubMed, Scopus, Web of Science, and Embase	PROSPERO: CRD42024548468	Newcastle–Ottawa Scale	Critically Low
Sun *et al*[33]	China	MACE after PCI, non-fatal MI after PCI, revascularization after PCI	ESC Heart Failure	24	10 164	RevMan (v:5.4)	Up to March 2023	Cochrane Library, PubMed, Embase, and Web of Science	PROSPERO: CRD42023387411	Newcastle-Ottawa Scale	Critically low
Wang *et al*[34]	China	MACE after PCI, MACE in ACS after PCI, ISR after PCI	Frontiers in Cardiovascular Medicine	17	22 467	STATA 17.0	Up to October 2023	PubMed, Embase, Cochrane Library, and Web of Science	PROSPERO: CRD42023467909	Newcastle-Ottawa Scale	Critically low
Yang *et al*[35]	China	HTN in general population	Clinical and Experimental Hypertension	7	35 848	Review Manager 5.3 and Stata 14.0	Up to July 2023	PubMed, EMBASE, and Web of Science	PROSPERO: CRD42023447674	Newcastle-Ottawa Scale	Critically low
						RevMan (v:5.1) and STATA 12.0	–	PubMed, Web of Science, and Embase	No	Newcastle-Ottawa Scale	Critically low
Cai *et al*[36]	China	ACD in HF, MACE in HF, CV mortality in HF	Frontiers in Endocrinology	19	44 275	STATA 15.0	Up to March 2025	PubMed, Cochrane, EMBASE, and Web of Science	CRD42024562063	Newcastle-Ottawa Scale	Critically low
Chen *et al*[37]	China	Risk of AF	Asia Pacific Journal of Clinical Nutrition	17	57 213	RevMan (v:5.3) and STATA 16.0	Up to August 2024	PubMed, Embase, Web of Science, Wanfang, and China National Knowledge Internet databases	PROSPERO: CRD42024581745	Newcastle-Ottawa Scale	Critically low
Dakota *et al*[38]	Indonesia	MACE in HTN, MI outcome in HTN, CV mortality in HTN	American Journal of Preventive Cardiology	20	451 455	Review Manager version 5.4	Up to November 2024	PubMed, Science direct, and Cochrane library	PROSPERO: CRD42025634989	Newcastle-Ottawa Scale	Critically low
Lai *et al*[39]	China	MACE in CAD, CV mortality in CAD, MI in CAD, revascularization in CAD	Medicine	23	50 793	STATA 14.0	Up to December 2024	Web of Science, PubMed, and Embase	No	Newcastle-Ottawa Scale	Critically low
Ren *et al*[40]	China	HTN	Frontiers in Endocrinology	36	1 021 533	STATA 17.0	–	PubMed, Embase, and the Cochrane Library	No	Newcastle-Ottawa Scale	Critically low
Zhang *et al*[43]	China	CVD events, MACCE, IHD, CV mortality	Cardiovascular Diabetology	50	7 239 790	RevMan (v:5.4) and R software 4.1.2	Up to December 2024	PubMed, EMBASE and the Cochrane Library	PROSPERO: CRD42024506648	Newcastle-Ottawa Scale	Critically low
Yu *et al*[42]	China	CV mortality in HF, rehospitalization in HF, MACE in HF	Biomolecules and Biomedicine,	12	20 639	RevMan (v:5.1) and STATA 12.0	Up to January 2024	PubMed, Web of Science, and Embase	No	Newcastle-Ottawa Scale	Critically low
Xu *et al*[41]	China	MACE in CHD, non-fatal MI in CHD, revascularization in CHD	Cardiovascular Diabetology	36	173 851	R software 4.4.1 and STATA 17.0	Up to March 2025	PubMed, the Cochrane Library, Web of Science, and Embase	PROSPERO: CRD420251018545	Newcastle-Ottawa Scale	Critically low

ACS, acute coronary syndrome; ACD, all-cause death; AF, atrial fibrillation; AMI, acute myocardial infarction; AMSTAR-2, A Measurement Tool to Assess Systematic Reviews 2; ASCVD, atherosclerotic cardiovascular disease; AHRQ, Agency for Healthcare Research and Quality; CAC, coronary artery calcification; CAD, coronary artery disease; CBM, Chinese Biomedical Literature Database; CCS, chronic coronary syndrome; CHD, coronary heart disease; CIN, contrast-induced nephropathy; CNKI, China National Knowledge Infrastructure; CV, cardiovascular; CVD, cardiovascular disease; EMBASE, Excerpta Medica Database; HF, heart failure; HTN, hypertension; IHD, ischemic heart disease; ISR, in-stent restenosis; MACCE, major adverse cardiac and cerebrovascular events; MACE, major adverse cardiovascular events; MI, myocardial infarction; NOS, Newcastle–Ottawa Scale; PAD, peripheral artery disease; PCI, percutaneous coronary intervention; PROSPERO, International Prospective Register of Systematic Reviews; QUADAS-2, Quality Assessment of Diagnostic Accuracy Studies-2; RevMan, Review Manager; TyG, triglyceride–glucose index.

### Data synthesis

For each CVO, we identified all eligible meta-analyses and extracted the primary pooled effect estimate (OR, RR, or HR), with its 95% CI and *I*^2^ heterogeneity statistic. When multiple meta-analyses addressed the same CVO, we selected the meta-analysis with the largest total sample size to avoid duplication of overlapping primary studies and ensure the most precise estimate. In cases in which two meta-analyses had comparable sample sizes, we prioritized the meta-analysis with the more recent search date or higher AMSTAR-2 quality rating. For CVOs for which only a single meta-analysis was available, that meta-analysis was used as the primary source of evidence.

We did not perform a quantitative reanalysis or meta-analysis of individual participant data. Instead, we synthesized results narratively, by CVO, organizing findings according to the specific outcome and how the TyG index was analyzed: categorical analysis (highest vs. lowest) or continuous analysis (per 1-unit increase). When heterogeneity (*I*^2^) exceeded 50%, we interpreted pooled estimates with caution and noted whether the authors of the original meta-analysis had explored potential sources of heterogeneity through subgroup or sensitivity analyses. Findings from meta-analyses with *I*^2^ > 75% and no reported explanation for heterogeneity were considered less reliable.

## Results

### Study selection

A total of 384 studies were identified through searches of PubMed, Web of Science, and Scopus from database inception to October 2025. After duplicates were removed, 286 studies were selected for title and abstract screening. After irrelevant studies were excluded and full texts were evaluated, 27 articles were selected for inclusion^[^12,14^]^ (Fig. 1).

**Figure 1.**
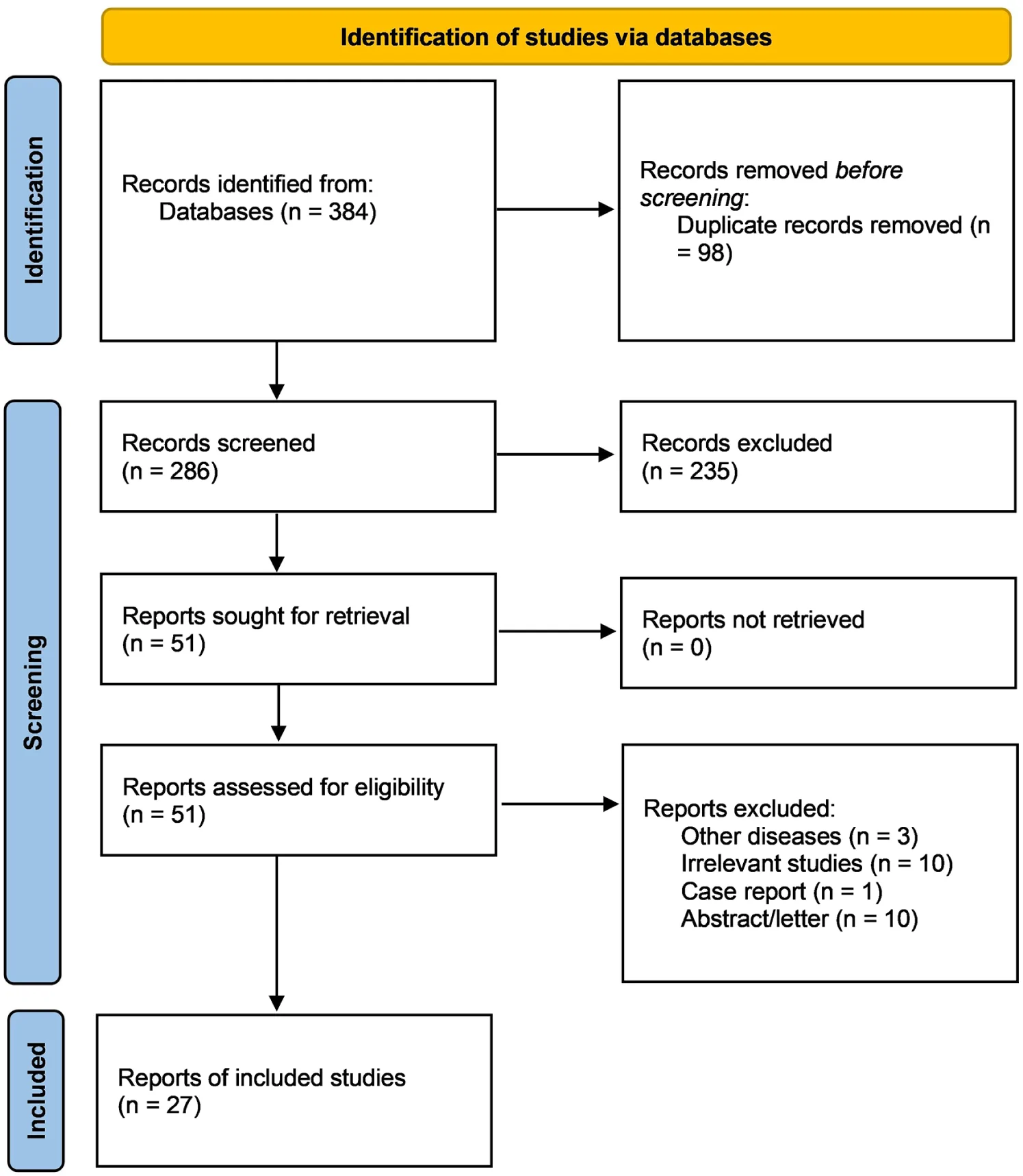
PRISMA flow diagram.

### Study characteristics

Table 1 presents the characteristics of the included studies. The studies included in this review were published between 2021 and 2025. The literature searches in these studies were conducted across different timeframes, with the earliest search extended from database inception to 5 January 2021, and the latest extended to March 2025. Most studies conducted their searches across multiple databases, including PubMed, Embase, Scopus, Web of Science, and the Cochrane Library. Total sample sizes varied substantially across studies, ranging from 886 to 7,239,790 participants. The meta-analyses originated from various countries: 20 studies from China^[^12,20–23,25,27,28,17,30,39–43^]^, three studies from Iran^[^14,29,32^]^, two studies from Indonesia^[^26,38^]^, one study from the USA[24], and one study from Taiwan[16]. Finally, 20 studies registered their study protocols in PROSPERO^[^12,14,21,23,16,17,25,27–29,26,32–35,36–38,41,43^]^, and seven studies did not register their protocols^[^20,22,24,30,39,40,42^]^. Furthermore, four studies had low-quality evidence^[^21,16,25,27^]^ and 23 studies had critically low-quality evidence based on the AMSTAR-2 checklist^[^12,14,20,22–24,17,26,28–30,32–43^]^.

### Summary of associations between the TyG Index and CVOs

An overview of the pooled associations between the TyG index and CVOs is presented in Tables 2–8. The results are separated into outcome-specific tables, including coronary, vascular, arrhythmic, composite, mortality, procedural/post-procedural, and ischemic heart disease outcomes.

**Table 2:** Association between the TyG index and coronary outcomes.

Outcome	TyG analysis	Effect size	*I*^2^
ASCVD	Highest vs. lowest	HR = 1.61 (1.29–2.01)	80%
ASCVD	Per 1-unit increase	HR = 1.28 (1.13–1.45)	61%
CAD	Highest vs. lowest	HR = 1.95 (1.47–2.58)	92%
CAD	Per 1-unit increase	HR = 1.39 (1.18–1.64)	89%
Coronary artery stenosis	Continuous	OR = 3.49 (1.71–7.12)	0%
MI, general population	Highest vs. lowest	HR = 1.36 (1.18–1.56)	35%
MI, ACS patients	Highest vs. lowest	HR = 1.58 (1.28–1.96)	0%
MI, CCS patients	Highest vs. lowest	HR = 3.80 (1.95–7.40)	0%
MI, CAD patients	Highest vs. lowest	HR = 1.72 (1.33–2.23)	0%
Non-fatal MI after PCI	Highest vs. lowest	HR = 2.02 (1.31–3.10)	0%
Non-fatal MI after PCI	Per 1-unit increase	HR = 2.57 (1.49–4.41)	63%
MI outcomes among HTN patients	Highest vs. lowest	HR = 1.55 (1.27–1.88)	0%
MI outcomes among HTN patients	Per 1-unit increase	HR = 0.84 (0.70–1.01)	0%
MI among CHD patients, diabetic	Highest vs. lowest	HR = 2.05 (1.52–2.77)	0%
MI among CHD patients, non-diabetic	Highest vs. lowest	HR = 2.46 (1.11–5.47)	59%

ACS, acute coronary syndrome; ASCVD, atherosclerotic cardiovascular disease; CAD, coronary artery disease; CCS, chronic coronary syndrome; CHD, coronary heart disease; HR, hazard ratio; HTN, hypertension; MI, myocardial infarction; OR, odds ratio; PCI, percutaneous coronary intervention; TyG, triglyceride-glucose index.

### Coronary outcomes

A higher TyG index was consistently associated with an increased risk of coronary outcomes. The strongest association was observed for coronary artery stenosis, for which each 1-unit increase in the TyG index was associated with 3.49-fold higher odds [OR = 3.49, 95% CI: 1.71–7.12, *P* < 0.01; *I*^2^ = 0%]. Among patients with chronic coronary syndrome (CCS), the highest TyG category was associated with a 3.80-fold higher risk of myocardial infarction (MI) (HR = 3.80, 95% CI: 1.95–7.40, *P* < 0.01; *I*^2^ = 0%). The pooled associations for coronary outcomes are summarized in Table 2.

### Vascular outcomes

An elevated TyG index was significantly associated with all vascular outcomes examined. In cross-sectional studies, the highest TyG category was associated with 2.01-fold higher odds of hypertension (HTN) compared with the lowest category (OR = 2.01, 95% CI: 1.47–2.76, *P* < 0.01; *I*^2^ = 99%). For peripheral arterial disease (PAD), the highest TyG quartile was associated with 94% higher odds compared with the lowest quartile (OR = 1.94, 95% CI: 1.49–2.54, *P* < 0.01; *I*^2^ = 85%). The pooled associations for vascular outcomes are summarized in Table 3.

**Table 3:** Association between the TyG index and vascular outcomes.

Outcome	TyG analysis	Effect size	*I*^2^
HTN, cohort studies	Highest vs. lowest	HR = 1.57 (1.25–1.96)	97%
HTN, cross-sectional studies	Highest vs. lowest	OR = 2.01 (1.47–2.76)	99%
HTN	Per 1-unit increase	HR = 1.36 (1.28–1.45)	69%
Arterial stiffness	Highest vs. lowest	OR = 1.83 (1.55–2.17)	68%
Arterial stiffness	Per 1-unit increase	OR = 1.51 (1.35–1.69)	82%
CAC	Highest vs. lowest	OR = 1.66 (1.51–1.82)	0%
CAC	Continuous	OR = 1.73 (1.36–2.20)	51%
CAC progression	Highest vs. lowest	OR = 1.66 (1.21–2.27)	0%
CAC progression	Continuous	OR = 1.47 (1.29–1.68)	41%
PAD	Continuous	OR = 1.60 (1.41–1.80)	69%
PAD	Highest vs. lowest	OR = 1.94 (1.49–2.54)	85%

CAC, coronary artery calcification; HR, hazard ratio; HTN, hypertension; OR, odds ratio; PAD, peripheral arterial disease; TyG, triglyceride-glucose index.

### Arrhythmic outcomes

A higher TyG index was significantly associated with atrial fibrillation (AF). For each 1-unit increase in the TyG index, the odds of AF increased by 80% (OR = 1.80, 95% CI: 1.50–2.17, *P* < 0.01; *I*^2^ = 88%). Among patients undergoing AF ablation, the highest TyG category was associated with 2.29-fold higher odds of AF recurrence (OR = 2.29, 95% CI: 1.14–4.58, *P* = 0.02; *I*^2^ = 93%). The pooled associations for arrhythmic outcomes are shown in Table 4.

**Table 4:** Association between the TyG index and arrhythmic outcomes.

Outcome	TyG analysis	Effect size	*I*^2^
AF	Highest vs. lowest	OR = 1.98 (1.35–2.91)	92%
AF	Per 1-unit increase	OR = 1.80 (1.50–2.17)	88%
AF recurrence after ablation	Highest vs. lowest	OR = 2.29 (1.14–4.58)	93%

AF, atrial fibrillation; OR, odds ratio; TyG, triglyceride-glucose index.

### Composite outcomes

An elevated TyG index consistently predicted a higher risk of major adverse cardiovascular events (MACE) across multiple populations. Among patients with acute coronary syndrome (ACS), the highest TyG category was associated with a 2.09-fold higher hazard of MACE (HR = 2.09, 95% CI: 1.68–2.62, *P* < 0.01; *I*^2^ = 87%). After acute myocardial infarction (AMI), a higher TyG index was associated with a 65% higher hazard of major adverse cardiac and cerebrovascular events (MACCE) (HR = 1.65, 95% CI: 1.34–2.04, *P* < 0.01; *I*^2^ = 65%). The pooled associations for composite outcomes are summarized in Table 5.

**Table 5:** Association between the TyG index and composite CVOs.

Outcome	TyG analysis	Effect size	*I*^2^
MACE, CHD patients, diabetic	Highest vs. lowest	HR = 1.98 (1.61–2.43)	87%
MACE, CHD patients, diabetic	Per 1-unit increase	HR = 1.57 (1.38–1.78)	71%
MACE, CAD patients	Highest vs. lowest	HR = 1.66 (1.47–1.88)	69%
MACE, ACS patients	Highest vs. lowest	HR = 2.09 (1.68–2.62)	87%
MACE, ACS patients	Per 1-unit increase	OR = 2.28 (1.44–3.63)	95%
MACE, CCS or stable CAD	Highest vs. lowest	HR = 1.24 (0.96–1.60)	85%
MACE, CCS or stable CAD	Per 1-unit increase	OR = 1.49 (1.20–1.83)	75%
MACE, post-PCI	Highest vs. lowest	HR = 2.04 (1.65–2.52)	77%
MACE, post-PCI	Per 1-unit increase	HR = 1.60 (1.48–1.73)	39%
MACE, HF patients	Per 1-unit increase	HR = 1.56 (1.32–1.85)	81%
MACE, HTN patients	Highest vs. lowest	HR = 1.90 (1.41–2.57)	88%
MACE, HTN patients	Per 1-unit increase	HR = 1.22 (1.11–1.35)	47%
MACCE after AMI	Highest vs. lowest	HR = 1.65 (1.34–2.04)	65%
MACCE after AMI	Per 1-unit increase	HR = 1.66 (1.15–2.40)	96%

ACS, acute coronary syndrome; AMI, acute myocardial infarction; CAD, coronary artery disease; CCS, chronic coronary syndrome; CHD, coronary heart disease; HF, heart failure; HR, hazard ratio; HTN, hypertension; MACE, major adverse cardiovascular events; MACCE, major adverse cardiac and cerebrovascular events; OR, odds ratio; PCI, percutaneous coronary intervention; TyG, triglyceride-glucose index.

### Mortality outcomes

The association between the TyG index and CVD mortality varied by population. Significant associations were observed among patients with HF, in whom the highest TyG category was associated with an 87% higher risk of CVD mortality (RR = 1.87, 95% CI: 1.42–2.47, *P* < 0.01; *I*^2^ = 57%), and among patients with HTN, in whom the highest TyG quartile was associated with a 72% higher hazard (HR = 1.72, 95% CI: 1.05–2.82, *P* < 0.01; *I*^2^ = 0%). However, in the general population, no significant association was found between the TyG index and CVD mortality (HR = 1.10, 95% CI: 0.82–1.47, *P* = 0.53; *I*^2^ = 76%). The pooled associations for mortality outcomes are summarized in Table 6.

**Table 6:** Association between the TyG index and cardiovascular mortality outcomes.

Outcome	TyG analysis	Effect size	*I*^2^
CVD mortality, diabetic patients	Highest vs. lowest	HR = 1.85 (1.16–2.95)	86%
CVD mortality, ACS patients	Highest vs. lowest	HR = 1.51 (1.12–2.02)	39%
CVD mortality, CAD patients	Highest vs. lowest	HR = 1.44 (1.00–2.06)	72%
CVD mortality, general population	Highest vs. lowest	HR = 1.10 (0.82–1.47)	76%
CVD mortality, HF patients	Highest vs. lowest	RR = 1.87 (1.42–2.47)	57%
CVD mortality, HF patients	Per 1-unit increase	HR = 1.14 (0.72–1.85)	85%
CVD mortality, HTN patients	Highest vs. lowest	HR = 1.72 (1.05–2.82)	0%

ACS, acute coronary syndrome; CAD, coronary artery disease; CVD, cardiovascular disease; HF, heart failure; HR, hazard ratio; HTN, hypertension; RR, relative risk; TyG, triglyceride-glucose index.

### Procedural and post-procedural outcomes

A higher TyG index was significantly associated with adverse outcomes following cardiovascular procedures. Among patients undergoing percutaneous coronary intervention (PCI), a higher TyG index was associated with 2.25-fold higher odds of contrast-induced nephropathy (CIN) (OR = 2.25, 95% CI: 1.82–2.77, *P* < 0.01; *I*^2^ = 35%). For revascularization outcomes, the strongest association was observed in patients with ACS, in whom the highest TyG category was associated with a 2.42-fold higher hazard (HR = 2.42, 95% CI: 1.64–3.57, *P* < 0.01; *I*^2^ = 82%). Regarding in-stent restenosis (ISR), each 1-unit increase in the TyG index was associated with 42% higher odds of ISR among patients with coronary heart disease (CHD) undergoing PCI (OR = 1.42, 95% CI: 1.26–1.59, *P* < 0.01; *I*^2^ = 13%). The pooled associations for procedural and postprocedural outcomes are summarized in Table 7.

**Table 7:** Association *between* the TyG index and procedural and postprocedural CVOs.

Outcome	TyG analysis	Effect size	*I*^2^
Revascularization, CAD patients	Highest vs. lowest	HR = 1.72 (1.33–2.23)	0%
Revascularization, ACS patients	Highest vs. lowest	HR = 2.42 (1.64–3.57)	82%
Revascularization, ACS patients	Per 1-unit increase	HR = 2.64 (2.08–3.35)	23%
Revascularization, CCS patients	Highest vs. lowest	HR = 1.30 (1.07–1.59)	0%
Revascularization, post-PCI	Highest vs. lowest	HR = 2.61 (1.47–4.66)	84%
Revascularization, post-PCI	Per 1-unit increase	HR = 2.06 (1.21–3.50)	90%
Revascularization, CHD patients, diabetic	Highest vs. lowest	HR = 2.52 (1.26–5.04)	91%
Revascularization, CHD patients, non-diabetic	Highest vs. lowest	HR = 2.09 (1.57–2.76)	0%
CIN after PCI	Continuous	OR = 2.25 (1.82–2.77)	35%
ISR, CHD patients post-PCI	Per 1-unit increase	OR = 1.42 (1.26–1.59)	13%
ISR, CHD patients post-PCI	Highest vs. lowest	OR = 1.69 (1.32–2.17)	0%
ISR, CCS patients post-PCI	Per 1-unit increase	OR = 1.37 (1.19–1.57)	0%
ISR, ACS patients post-PCI	Per 1-unit increase	OR = 1.48 (1.19–1.85)	0%

ACS, acute coronary syndrome; CAD, coronary artery disease; CCS, chronic coronary syndrome; CHD, coronary heart disease; CIN, contrast-induced nephropathy; HR, hazard ratio; ISR, in-stent restenosis; OR, odds ratio; PCI, percutaneous coronary intervention; TyG, triglyceride-glucose index.

**Table 8:** Association between the TyG index and ischemic heart disease outcomes.

Outcome	TyG analysis	Effect size	*I*^2^
IHD, diabetic patients	Highest vs. lowest	HR = 2.20 (1.71–2.83)	0%
IHD, non-diabetic patients	Highest vs. lowest	HR = 1.57 (1.30–1.89)	81%

HR, hazard ratio; IHD, ischemic heart disease; TyG, triglyceride-glucose index.

### Ischemic heart disease

Among patients with diabetes, the highest TyG category was associated with a 2.20-fold higher hazard of ischemic heart disease (IHD) (HR = 2.20, 95% CI: 1.71–2.83, *P* < 0.01; *I*^2^ = 0%). Among patients without diabetes, the highest TyG category was associated with a 57% higher hazard of IHD (HR = 1.57, 95% CI: 1.30–1.89, *P* < 0.01; *I*^2^ = 81%). The pooled associations for ischemic heart disease outcomes are summarized in Table 8.

## Discussion

In this umbrella review of 27 meta-analyses, higher TyG index values were generally associated with adverse CVOs in general and high-risk populations. Associations were observed across coronary, vascular, arrhythmic, composite, and postprocedural outcomes. However, findings for cardiovascular mortality varied across populations and were not statistically significant in the general population. Although the overall direction of the associations was broadly consistent, substantial heterogeneity and the low or critically low methodological quality of the included meta-analyses limit confidence in the magnitude, precision, and generalizability of the pooled estimates.

To reduce duplication arising from overlapping meta-analyses, we selected one meta-analysis per outcome using predefined criteria based on sample size, recency, and methodological quality. Although this approach reduced double-counting, it may have excluded relevant evidence from smaller meta-analyses, and overlap among primary studies was not formally quantified.

The TyG index, calculated from fasting triglyceride and glucose concentrations, has emerged as a reliable surrogate marker of IR, demonstrating strong correlations with both the hyperinsulinemic-euglycemic clamp and the homeostasis model assessment of insulin resistance (HOMA-IR)[44].

Compared with insulin-based indices, the TyG index offers superior practicality and reproducibility in large-scale population studies, which may partly explain its increasingly consistent associations with CVOs across diverse cohorts^[^44–47^]^. Previous individual meta-analyses have reported associations between an elevated TyG index and selected cardiovascular endpoints, such as CAD, hypertension, and major adverse cardiovascular events. However, these studies were typically confined to specific populations or single outcomes^[^12,13,31^]^. By integrating evidence across a wide range of cardiovascular phenotypes, including ASCVD, MI across multiple clinical contexts, arterial stiffness, coronary artery calcification, AF, heart failure outcomes, and revascularization, this umbrella review demonstrates that the cardiovascular implications of IR captured by the TyG index are broad, systemic, and clinically meaningful.

Several interrelated biological mechanisms may explain the observed associations. First, IR induces endothelial dysfunction through impaired insulin-mediated nitric oxide synthesis and enhanced endothelin-1 activity, leading to vasoconstriction, reduced arterial compliance, and increased arterial stiffness[48]. This mechanism aligns with the robust associations observed between the TyG index and arterial stiffness, hypertension, and coronary artery calcification. Increased arterial stiffness, in turn, contributes to elevated systolic blood pressure, left ventricular hypertrophy, and impaired coronary perfusion, thereby accelerating the development of ASCVD and CAD^[^49,50^]^.

Second, IR is closely linked to atherogenic dyslipidemia, characterized by elevated triglyceride levels, increased levels of small, dense low-density lipoprotein particles, and reduced high-density lipoprotein cholesterol levels^[^51,52^]^. This lipid profile promotes lipid accumulation within the arterial wall, foam cell formation, and plaque progression[51].

The associations observed in this review between the TyG index and coronary artery stenosis, CAC presence and progression, PAD severity, and IHD are consistent with lipid-driven atherogenic pathways. Third, chronic low-grade inflammation represents a key mechanistic bridge between IR and CVD[53].

Insulin-resistant states are associated with increased circulating levels of proinflammatory cytokines, increased oxidative stress, and NLRP3 inflammasome activation, all of which contribute to endothelial injury, plaque instability, and thrombogenesis[48]. These inflammatory processes may partially explain the heightened risks of MI, MACE, and MACCE observed in patients with an elevated TyG index. In addition, IR adversely affects myocardial structure and function through altered substrate utilization, mitochondrial dysfunction, and myocardial fibrosis^[^54–56^]^. These processes may underlie the observed associations between the TyG index and HF-related outcomes, rehospitalization, and cardiovascular mortality in populations with HF. Similarly, metabolic dysfunction and inflammation may promote atrial structural remodeling and electrical instability, providing a plausible explanation for the associations between the TyG index and AF incidence, recurrence after ablation, and post-procedural AF[57]. Taken together, these findings reinforce the concept that the TyG index captures a central pathophysiological axis linking metabolic dysfunction to CVD[58]. Rather than being confined to a single outcome, IR, as reflected by an elevated TyG index, appears to influence the entire CVD continuum, from subclinical vascular damage to overt clinical events and adverse long-term outcomes.

The TyG index has practical appeal because it is inexpensive and can be calculated from routinely measured fasting glucose and triglyceride concentrations without requiring insulin measurement^[^44,59^]^. Associations with in-hospital or ICU mortality and with major adverse cardiovascular events (MACE) among patients with CAD support further investigation of its prognostic value^[^39,60^]^. However, these studies do not establish that TyG-guided surveillance, preventive interventions, or treatment improve clinical outcomes. Accordingly, the TyG index should currently be regarded as a potential adjunct to, rather than a replacement for, established cardiovascular risk assessment tools.

Despite the consistent associations observed in this umbrella review, the clinical application of the TyG index remains limited by the absence of a standardized cutoff value. Most included studies used population-specific tertiles, quartiles, or median values rather than fixed thresholds. Proposed cutoff values of approximately 8.5–9.0 were derived from heterogeneous settings, including NAFLD detection using a cutoff of 8.5[61], hepatic steatosis and fibrosis using a cutoff of approximately 8.7[62], and coronary or other adverse outcomes at values that sometimes exceeded 9.0^[^63,64^]^. These thresholds should not, therefore, be generalized across populations or CVOs, and population- and outcome-specific validation is required.

Compared with the hyperinsulinemic-euglycemic clamp and HOMA-IR, the TyG index is more practical because it is calculated from routinely available fasting glucose and triglyceride measurements and does not require insulin testing. However, its incremental predictive value beyond that of established cardiovascular risk models remains uncertain. The TyG index may therefore be considered an adjunctive tool for screening and risk stratification, particularly in resource-limited settings, but it should not replace validated risk scores. Prospective studies are needed to determine whether incorporating the TyG index into clinical practice improves risk prediction or clinical outcomes before routine implementation can be recommended.

A critical consideration in this umbrella review is the substantial heterogeneity observed across many pooled estimates. Several outcomes had *I*^2^ values exceeding 80%, and in some cases 90%. Such high heterogeneity indicates considerable variability between studies; it also suggests that the pooled effect estimates may not represent a single underlying true effect. This variability likely reflects multiple sources, including differences in study populations, TyG index cutoff values and categorization methods, outcome definitions, follow-up duration, and adjustment for confounding variables. Study populations also varied widely, ranging from the general population to high-risk groups, including patients with diabetes, hypertension, or established CVD.

From a clinical perspective, this degree of heterogeneity has important implications. Although the consistent direction of association across most outcomes supports the role of the TyG index as a marker of increased cardiovascular risk, the wide variability in effect estimates limits the direct applicability of specific quantitative estimates for individual risk prediction. Clinicians should therefore be cautious when interpreting the magnitude of risk associated with a given TyG index value. Reported effect estimates may differ substantially according to patient characteristics and clinical context. This is particularly relevant when using the TyG index for risk stratification, where standardized thresholds and consistent effect estimates are essential for decision-making.

High heterogeneity also reduces confidence in the precision and reproducibility of pooled estimates. The observed associations may therefore be influenced by unmeasured confounding or methodological differences across studies. Accordingly, the findings of this umbrella review should be interpreted as evidence of a consistent association rather than as a precise quantification of risk. The TyG index may be most useful as an adjunctive marker to identify individuals at elevated cardiometabolic risk rather than as a stand-alone tool for clinical decision-making. Future studies with standardized designs, uniform definitions, and population-specific analyses are needed to reduce heterogeneity and improve the clinical applicability of these findings.

Although the TyG index shows promise as an additional risk marker, clinical decision-making should remain guided by established cardiovascular risk assessment tools and clinical guidelines. The TyG index should not replace these tools. Rather, it should be considered an adjunctive biomarker that may provide added value in specific clinical scenarios, particularly when metabolic abnormalities are present or advanced testing is unavailable. Clinicians should balance the simplicity and low cost of the TyG index against the lack of standardized cutoffs and the limited evidence that TyG-guided management improves outcomes. Until prospective studies establish clear clinical protocols, the TyG index is best used as an awareness tool to identify individuals who may benefit from more comprehensive cardiovascular risk assessment and lifestyle modification, in line with current preventive guidelines.

### Limitations

This umbrella review has several limitations that should be acknowledged. First, the findings are dependent on the quality and methodology of the included systematic reviews and meta-analyses, and residual bias from primary studies cannot be excluded. Second, substantial heterogeneity was observed in many pooled estimates, likely reflecting differences in study design, populations, TyG index cutoff values, outcome definitions, follow-up duration, and covariate adjustment, which may limit the comparability and generalizability of the results. Performing subgroup analyses to formally evaluate the sources of this heterogeneity is not typically within the scope of an umbrella review, as it would require outcome-specific subgroup analyses and substantially increase the complexity and length of the article. Nevertheless, we acknowledge this as a limitation of our study.

Third, the AMSTAR-2 assessment revealed that most included meta-analyses were rated as being of critically low quality, and the remaining four as being of low quality. This finding substantially affects the reliability of our conclusions. Although the direction of associations was consistent across most outcomes, the critically low methodological quality of the underlying evidence means that the pooled effect estimates may be biased or overestimated, and the certainty of the evidence is low. Therefore, our findings should be interpreted with considerable caution and highlight the urgent need for more rigorously designed and conducted meta-analyses in this field.

Fourth, most of the included meta-analyses were based on observational studies, precluding causal inference and leaving the associations vulnerable to residual confounding and reverse causation. Fifth, reporting inconsistencies across reviews limited our ability to uniformly compare effect measures, perform formal reanalyses, or assess dose-response relationships across outcomes. Sixth, publication bias and small-study effects could not be consistently evaluated across outcomes, and selective reporting may have influenced the strength of the observed associations. Finally, most evidence originated from specific geographic regions and high-risk populations, which may restrict the applicability of the findings to broader or underrepresented populations.

## Conclusion

Available evidence suggests that higher TyG index values are associated with an increased risk of several CVOs across general and high-risk populations. However, confidence in the magnitude, precision, and generalizability of these associations is limited by substantial between-study heterogeneity, inconsistent TyG index definitions and cutoff values, the predominantly observational nature of the underlying evidence, and the low or critically low methodological quality of the included meta-analyses. Therefore, these findings should be interpreted as evidence of association rather than causality and do not establish a definitive dose-response relationship or a universally applicable TyG index threshold.

Given its simplicity, low cost, and availability through routine laboratory measurements, the TyG index may serve as an adjunctive marker within established cardiovascular risk assessment frameworks, particularly for individuals with metabolic abnormalities. Nevertheless, it should not currently be used as a standalone predictor or as the basis for treatment decisions. Well-designed prospective studies using standardized definitions and cutoff values are required to determine its incremental predictive value beyond established risk factors, validate its applicability across diverse populations, and assess whether TyG-guided strategies improve clinical outcomes.

## Data Availability

The datasets generated and/or analyzed during this study are available from the corresponding author upon reasonable request.

## Research registration unique identifying number (UIN)

PROSPERO (CRD42024564660).

## Artificial intelligence (AI) declaration

This study complies with the TITAN Guidelines 2025 on the use of artificial intelligence (AI) in academic writing[65]. AI tools, including ChatGPT (OpenAI), were used exclusively to assist with correcting grammar, spelling, and punctuation, and with improving the fluency and readability of the manuscript. No AI tools were involved in data analysis, interpretation, or the creation of original scientific content. All intellectual contributions, critical analyses, and final decisions were made solely by the authors.
